# Genetic Contributions to Alzheimer’s Disease and Frontotemporal Dementia in Admixed Latin American Populations

**DOI:** 10.21203/rs.3.rs-5462510/v1

**Published:** 2025-05-06

**Authors:** Juliana Acosta-Uribe, Stefanie D. Piña Escudero, J. Nicholas Cochran, Jared W. Taylor, P. Alejandra Castruita, Caroline Jonson, Erin A. Barinaga, Kevin Roberts, Alexandra R. Levine, Dawwod S. George, José Alberto Avila-Funes, María I. Behrens, Martin A. Bruno, Luis I. Brusco, Nilton Custodio, Claudia Duran-Aniotz, Francisco Lopera, Diana L. Matallana, Andrea Slachevsky, Leonel T. Takada, Lina M. Zapata-Restrepo, Dafne E. Durón-Reyes, Elisa de Paula França Resende, Luisa F. Gómez, Nancy Gelvez, M. Beatriz Bistue, Maria E. Godoy, Marcelo A. Maito, Shireen Javandel, Bruce L. Miller, Mike A. Nalls, Hampton Leonard, Dan Vitale, Sara Bandres-Ciga, Mathew J. Koretsky, Andrew B. Singleton, Caroline B. Pantazis, Victor Valcour, Agustin Ibañez, Kenneth S. Kosik, Jennifer S. Yokoyama, Multi-Partner Consortium to Expand Dementia Research in Latin America (ReDLat)

**Affiliations:** University of California, Santa Barbara; Global Brain Health Institute; HudsonAlpha Institute for Biotechnology; HudsonAlpha Institute for Biotechnology; University of California, San Francisco; University of California, San Francisco; HudsonAlpha Institute for Biotechnology; HudsonAlpha Institute for Biotechnology; University of California, San Francisco; University of California, Santa Barbara; Instituto Nacional de Ciencias Médicas y Nutrición Salvador Zubirán; University of Chile; Universidad Católica de Cuyo; National Scientific and Technical Research Council; Instituto Peruano de Neurociencias; Adolfo Ibáñez University; University of Antioquia; Pontificia Universidad Javeriana; University of Chile; Hospital das Clínicas da Faculdade de Medicina da Universidade de São Paulo; Fundación Valle del Lili; Instituto Nacional de Ciencias Médicas y Nutrición Salvador Zubirán; Universidade Federal de Minas Gerais; Instituto de Genética Humana; Instituto de Genética Humana; Universidad Católica de Cuyo; Adolfo Ibáñez University; Adolfo Ibáñez University; Global Brain Health Institute; Global Brain Health Institute; Data Tecnica International (United States); Data Tecnica International (United States); Data Tecnica International (United States); Center for Alzheimer’s and Related Dementias (CARD); Data Tecnica International (United States); Center for Alzheimer’s and Related Dementias (CARD); Center for Alzheimer’s and Related Dementias (CARD); Global Brain Health Institute; Adolfo Ibáñez University; University of California, San Francisco; Global Brain Health Institute

## Abstract

Latin America’s diverse genetic landscape provides a unique opportunity to study Alzheimer’s disease (AD) and frontotemporal dementia (FTD). The Multi-Partner Consortium to Expand Dementia Research in Latin America (ReDLat) recruited 2,162 participants with AD, FTD, or as healthy controls from six countries: Argentina, Brazil, Chile, Colombia, Mexico, and Peru. Participants underwent genomic sequencing and population structure analyses were conducted using Principal Component Analysis and ADMIXTURE. The study revealed a predominant mix of American, African, and European ancestries, with an additional East Asian component in Brazil. Variant curation identified 17 pathogenic variants, pathogenic *C9orf72* expansion, and 44 variants of uncertain significance. Seventy families showed autosomal dominant inheritance, with 48 affected by AD and 22 by FTD. This represents the first large-scale genetic study of AD and FTD in Latin America, highlighting the need to consider diverse ancestries, social determinants of health, and cultural factors when assessing genetic risk for neurodegenerative diseases.

## Introduction

Latin America is home to a unique blend of Indigenous American, European, and African ancestries, thereby possessing a diverse admixed genetic landscape that emerged in the 1500s from the conquest of the Americas and the slave trade^1^. Significant East Asian immigration that occurred during the early 20th century has further contributed to the continent’s population diversity observed today^2,3^. This admixture harbors a spectrum of novel genetic variants, including some that may modulate susceptibility to neurodegenerative diseases like Alzheimer’s disease (AD) and frontotemporal dementia (FTD) or may result in a higher allelic frequency of known risk-conveying variants for neurodegeneration^4^. Studying these populations, with their complex genetic architecture, offers an invaluable resource for understanding neurodegenerative diseases.

Assessing admixed populations is particularly interesting because admixture introduces a rich heterogeneity of alleles, which can be crucial for understanding genetic risk. For instance, in the Colombian population, researchers from a single center in Medellín have identified 13 different pathogenic *PSEN1* variants from different ancestral backgrounds^4^. This contrasts with the nine independent variants described in a screening study from nine centers throughout the Iberian Peninsula^5^. The presumptive excess of *PSEN1* variants may have become fixed in a relatively small effective population due to the antimicrobial benefits of high beta-amyloid levels^4^. Additionally, in the Peruvian population, a recent genome-wide association study and functional analysis suggested that the *NFASC* gene, located on chromosome 1, is associated with AD. The *NFASC* locus showed Significant contributions from both European and African ancestries^6^. This finding emphasizes the crucial role of relatively recent founder effects among diverse ancestral backgrounds in uncovering novel variation and understanding the complex genetic foundations of AD.

Due to cultural, religious, and historical factors, Latin American families have traditionally been large. Government policies during the 1960s and 1970s further encouraged population growth, resulting in an average family size of six children^7^,^8^. Additionally, geographic and socioeconomic conditions often led to extended families residing in proximity^7^,^9^. This unique demographic structure provides an exceptional opportunity to study founder effects in large families by tracing the lineage and impact of specific genetic variants across generations. For example, the *PSEN1* E280A (Glu280Ala) variant in Colombia can be traced back approximately 500 years to the time of the Spanish invasion. In the large families that descend from the original carrier, the variant became fixed and spread widely among numerous distant relatives. This historical and genetic tracing provides valuable insights into how genetic drift occurs over time in small populations, increasing the likelihood of identifying both risk and protective genetic variants^10–12^.

The scientific community from the region recognized the immense potential of this approach and formed the Multi-Partner Consortium to Expand Dementia Research in Latin America (ReDLat)^13^. This consortium fosters collaboration among researchers, clinicians, and institutions across six Latin American countries and the United States to leverage the unique genetic diversity and demographic characteristics that influence AD and FTD in Latin American populations. ReDLat aims to highlight the unique genetic diversity and demographic features of these populations associated with neurodegenerative diseases of cognition^14^. Ultimately, the consortium’s work seeks to refine diagnostic approaches, develop targeted therapeutic interventions, and Significantly enhance our understanding of dementia across Latin America^13^.

This paper is the first genetic report from this consortium, focusing on AD and FTD in admixed Latin American participants, with a particular emphasis on families. Our research efforts aim to identify genetic variants associated with these neurodegenerative diseases in the region and provide insights into some of the clinical presentations observed within the studied families, thereby enhancing our understanding of AD and FTD across this vast and diverse population. By expanding the genomic dataset in the coming years, we aim to deepen our understanding of the genetic underpinnings and clinical presentations of these neurodegenerative diseases across diverse Latin American populations, enhancing the potential for targeted interventions and therapies.

## Results

### A. Characterization of the population

**Figure 1:**
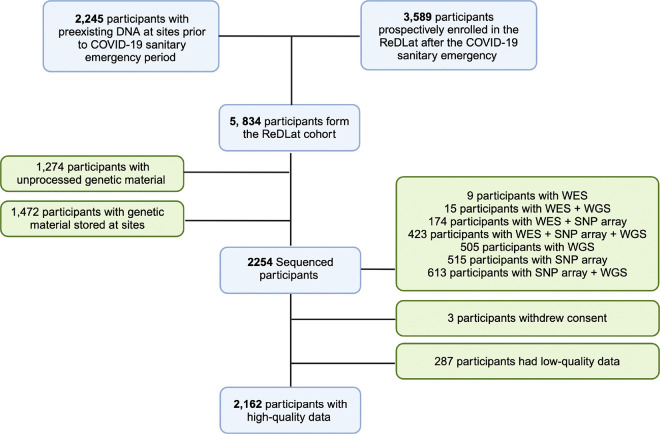
Assembly of ReDLat genomic dataset. WES: whole exome sequencing. WGS: whole genome sequencing. SNP: single nucleotide polymorphism

To investigate the genetic landscape of neurodegeneration in Latin America we recruited patients with mild to moderate AD or FTD, along with healthy controls from ten memory clinics across six Latin American countries: Argentina, Brazil, Chile, Colombia, Mexico, and Peru. Recruitment occurred in two phases due to the COVID-19 pandemic, which delayed the launch of ReDLat’s prospective enrollment, originally planned for 2020. Despite the differences in timing, the inclusion criteria for both the retrospective and prospective cohorts were identical. As of August 17, 2024, a total of 5,834 participants had been enrolled in the study [Figure 1].

By the time of manuscript submission, PCR-free whole genome sequencing (WGS), whole exome sequencing (WES), and/or single nucleotide polymorphism (SNP) array genotyping had been performed in 2,254 participants from both cohorts. After thorough quality control, genomic data from 2,162 individuals were retained for analysis. The final dataset included 658 participants with SNP array data, of whom 174 also had WES; 1,495 participants with WGS; and 9 individuals with only WES [Figure 1]. All participants underwent medical and neuropsychological evaluation. Among those with high-quality genomic data, 999 were diagnosed with AD, 381 with FTD, and 755 were classified as cognitively healthy at the time of assessment. The dataset also included eight participants with mild cognitive impairment and 19 individuals with other neuropsychiatric diagnoses, such as Parkinson’s disease, Lewy body dementia, atypical parkinsonism, neurodevelopmental disorders, cerebellar ataxia, brain tumor, cognitive impairment associated with non-brain cancer, vascular dementia, severe depressive disorder, bipolar disorder, obstructive sleep apnea, and chronic traumatic encephalopathy. These individuals were members of recruited families and had a relative enrolled in the study with AD- or FTD-related dementia.

As expected, a higher percentage of participants with FTD were under 65 years of age compared to those with AD (22.3% vs. 13.9%). Furthermore, the percentage of female participants was higher in the AD group (66.6%) compared to the FTD group (52.8%). Those in the AD group also showed a higher proportion of individuals who are heterozygous (41.5%) and homozygous (8.6%) for the *APOE* ε4 allele. [Table 1] The distribution of *APOE* alleles of unrelated individuals varies per country as shown in Supplementary Table 1. Homozygous *APOE* ε2 carriers were observed only in Colombia (0.2%) and Brazil (1%). Conversely, the highest numbers of *APOE* ε4 alleles were found in Argentina and Colombia (39% in both), although Colombia has 1% more homozygous carriers than Argentina. It is worth noting that these numbers may not be fully representative due to the Colombian sample size being considerably larger. As larger numbers of samples from all regions are collected, allele frequency estimates will be further refined.

### B. Genetic Ancestry

To assess genetic ancestry similarity among our samples, we initially generated a merged ReDLat dataset that included 2,153 participants with WGS or SNP array data that passed the concordance analysis. We then used WGS from the 1000 Genomes Project (1000GP) as reference populations to estimate the global ancestry of the participants, employing Principal Component Analysis (PCA) and ADMIXTURE software to estimate global ancestry. We used the 1000GP cohort to identify variants with allelic frequency >10% and in linkage equilibrium that were present in both the 1000GP and the merged ReDLat dataset, resulting in a total of 226,524 variants used for ancestry analyses.

**Figure 2:**
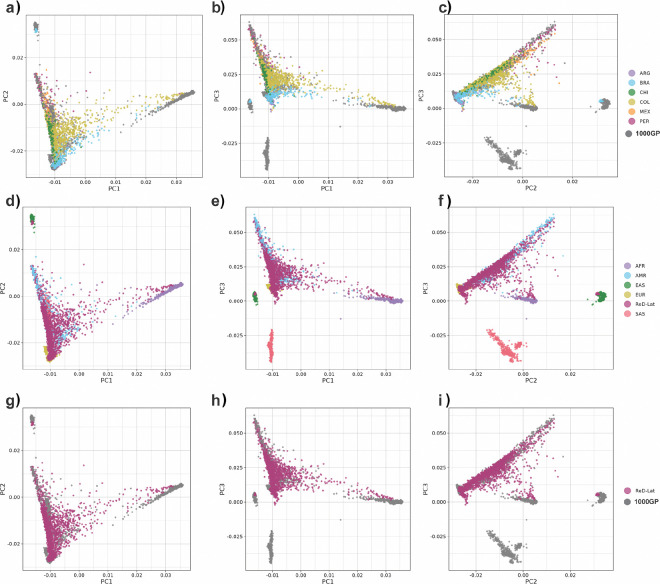
Principal component analysis of the ReDLat dataset and individuals from the 1000 Genome project (1000GP). The 1000 Genomes project includes African (AFR), European (EUR), South Asian (SAS), East Asian (EAS) and Admixed American (AMR) individuals. ReDLat sub-cohorts include individuals from Argentina (ARG), Brazil (BRA), Chile (CHI), Colombia (COL), Mexico (MEX) and Peru (PER). PC: Principal component. a) PC1 vs. PC2. Genomes from ReDLat are colored by sub-cohort, while all 1000GP genomes are shown in a single color. b) PC1 vs. PC3. Genomes from ReDLat are colored by sub-cohort, while all 1000GP genomes are shown in a single color. c) PC2 vs. PC3. Genomes from ReDLat are colored by sub-cohort, while all 1000GP genomes are shown in a single color. d) PC1 vs. PC2. Genomes from 1000GP are colored by sub-cohort, while all ReDLat genomes are shown in a single color. e) PC1 vs. PC3. Genomes from 1000GP are colored by sub-cohort, while all ReDLat genomes are shown in a single color. f) PC2 vs. PC3. Genomes from 1000GP are colored by sub-cohort, while all ReDLat genomes are shown in a single color. g) PC1 vs. PC2 of ReDLat and 1000GP genomes. h) PC1 vs. PC3 of ReDLat and 1000GP genomes. i) PC1 vs. PC2 of ReDLat and 1000GP genomes.

PCA [Figure 2, and Supplementary Figure 1] reveals that the ReDLat dataset shows substantial overlap with the American populations (AMR) sampled by the 1000GP, which also included Colombian, Mexican, and Peruvian participants. Though a substantial number of participants overlap with European (EUR) cohorts, there were 21 individuals clustering with the East Asian (EAS) population, suggesting recent EAS descent subgroups within ReDLat.

When analyzing the data by country, participants from Peru, Mexico, Chile, and Argentina exhibit minimal variation along Principal Component 1 (PC1), which is associated with African ancestry. In contrast, there is greater variation along Principal Component 2 (PC2), which is associated with Amerindian ancestry. This variation is particularly pronounced among participants from Mexico and Peru, who have individuals with a majority of their ancestry being Amerindian. These findings suggest a predominant two-way ancestry pattern in these populations. There is clear overlap between Argentinian and Brazilian samples with the European (EUR) cohort; however, the Brazilian samples show Significant variation along PC1, highlighting the African component of the sample. While most Brazilian samples are distributed primarily between African (AFR) and EUR populations, a small subset clusters with EAS, indicating a distinct ancestral subgroup within Brazil. Colombia displays a clear three-way admixture pattern, as evidenced by its wide distribution along both PC1 and PC2. Additionally, Peruvian and Mexican samples from ReDLat exhibit clear overlap with their 1000GP counterparts, while ReDLat Colombian samples showed greater diversity than those in 1000GP [Figure 2]. This increased diversity is likely due to ReDLat’s broader sampling across multiple regions of Colombia. Overall, the PCA analysis confirms that the ReDLat cohort accurately represents the different historical admixture patterns previously described in the corresponding countries^1^.

**Figure 3:**
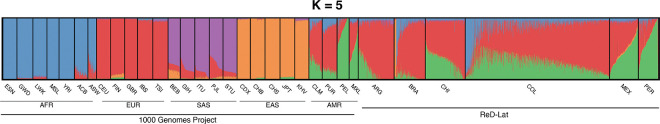
Global ancestry proportions of the ReDLat cohort represented by ADMIXTURE Q. values assuming 5 ancestral populations (K) The 1000 Genomes project includes African (AFR): GWD: Gambian in Western Divisions in the Gambia, LWK: Luhya in Webuye, MSL: Mende in Sierra Leone, YRI: Yoruba in Ibadan, Nigeria, ACB: African Caribbean in Barbados, ASW: African Ancestry in Southwest US. European (EUR): CEU: Utah Residents (CEPH) with Northern and Western European Ancestry, FIN: Finnish in Finland, GBR: British in England and Scotland, IBS: Iberian Population in Spain, TSI: Tuscany in Italia. South Asian (SAS): BEB: Bengali in Bangladesh, GHI: Gujarati Indians in Houston, ITU: Indian Telugu in the U.K., PJL: Punjabi in Lahore, STU: Sri Lankan Tamil in the UK. East Asian (EAS): CDX: Chinese Dai in Xishuangbanna, China, CHB: Han Chinese in Beijing, CHS: Han Chinese South, JPT: Japanese, Kyushu, KHV: Kinh Vietnamese. Admixed American (AMR): CLM: Colombian from Medellin, PUR: Puerto Rican from Puerto Rico. ReDLat subcohorts: ARG: Argentina, BRA: Brazil, CHI: Chile, COL: Colombia, MEX: Mexico, PER: Peru

To calculate global ancestry proportions (Q-values), which are adjusted p-values accounting for multiple testing and controlling the false discovery rate, we projected the ReDLat samples onto the 1000GP dataset ADMIXTURE results at multiple clustering values. [Supplementary Figure 2]. At K=5, where K represents the number of ancestral populations in the clustering analysis, we observed a continental separation of ancestral origins and were able to differentiate the Amerindian component. [Figure 3] Peru is the only country where Amerindian ancestry exceeds European ancestry, followed by Mexico, where these two ancestries show similar distribution. In Argentina, Brazil, Colombia, and Chile, European ancestry is the most prevalent among the participants, with a median value of 86.9% (Mean value of 79.7%, Standard deviation 17.5) for Argentina and 84.2% (Mean value of 73.8%, Standard deviation 26.9) for Brazil. African ancestry is present in Colombia and Brazil at lower levels; we observe a continuum of this ancestry, with individuals having over 90% and 75% African descent, down to the mean levels for both countries (around 10%), suggesting ongoing admixture over generations. In contrast, this continuum is not observed in the EAS component of the Brazilian samples, suggesting a recent diaspora without intercontinental admixture. [Supplementary Figure 3]

### C. Variant pathogenicity analysis

To identify Mendelian forms of neurodegeneration in our cohort, we analyzed data from 1,678 participants who had high-quality WGS or WES data to detect pathogenic variants. Following standard practices in complex systems analysis, we employed both “bottom-up” and “top-down” approaches.

Our bottom-up approach was a “gene-to-family” search, in which we initially assessed the genes most commonly associated with adult-onset neurodegeneration for pathogenic variants (see Methods). We identified a total of 17 pathogenic variants, a pathogenic *C9orf72* expansion, and 44 variants of uncertain significance (VUS). [Table 2 and Supplementary Table 2]. We note that the *C9orf72* expansions were identified using ExpansionHunter software from PCR-free WGS but were not explicitly confirmed by Southern blot, although this predictive tool is very accurate^15^.

In families with AD, we identified several previously described *PSEN1* variants: c.356C>T (p.Thr119Ile), c.415A>G (p.Met139Val) c.428T>C (p.Ile143Thr) and c.519G>T (p.Leu173Phe)^4,16,17^ [Supplementary Figure 4]. Notably, Thr119Ile and Ile143Thr are of European origin and exhibit identity by descent among Colombian carriers for Ile143Thr and between Colombian and Argentine carriers for Thr119Ile^4^. In contrast, the *PSEN1* c.415A>G (p.Met139Val) variant, identified in a large family from Argentina, was determined to be of Amerindian origin using the local ancestry inference software RFMix (see Methods). This haplotype was also present in two individuals from the 1000 Genomes Project, originating from Peru and Colombia [Supplementary Figure 5]. The *PSEN1* Met139Val variant has been reported across diverse ancestral backgrounds^18–20^ and is associated with either AD or atypical dementia, characterized by amnestic and behavioral symptoms. These symptoms may also include spastic paraparesis, psychosis, seizures, and myoclonus^21^. [See Supplementary Note 1 for the clinical description of this family].

In families with FTD, the gene most commonly associated with hereditary forms of the illness in our cohort was *GRN*, followed by *MAPT* [See Supplementary Notes 2 and 3 for the clinical description of these families]. Pathogenic *C9orf72* repeat expansions were observed in several families from geographically non-adjacent areas. The variants in *GRN*, *MAPT*, *C9orf72,* and *TARDBP* that were present in more than one individual were all of European descent [Table 3]. The pedigrees of families with pathogenic variants are shown in the Supplementary Figures 6–9.

After the initial analysis of these primary genes, we expanded our search to include secondary genes associated with adult-onset neurodegeneration. Utilizing the OMIM database, we identified a set of genes where single nucleotide variants or short insertions/deletions could be disease-causing [Supplementary Table 3]^19,22–24^. In our analysis, we found four additional pathogenic variants. [Supplementary Table 4 and 5] Three of these variants are present in families with autosomal dominant disease and are located in the *PRNP* and *NOTCH3* genes. Most notably, we identified an FTD patient without motor symptoms carrying a pathogenic variant in *SOD1* c.388G>A (p.Phe21Leu) which was previously reported in another FTD patient from the same geographical region and is believed to have originated an Amerindian haplotype^4^.

**Figure 4:**
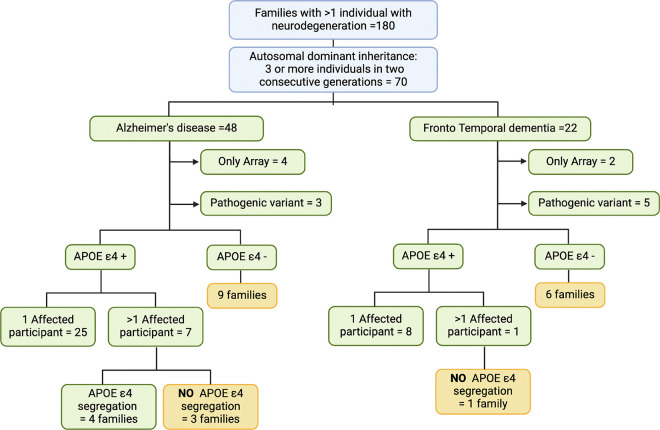
Cosegregation analysis of *APOE* ε4and neurodegeneration in families with multiple affected individuals

In contrast, our top-to-bottom approach consisted of a “family-to-gene” search, where we analyzed the pedigrees of all recruited participants with WES or WGS data available (766 individuals grouped in 592 families). After excluding individuals recruited as “healthy”, we identified 426 independent families and classified them based on the presence of affected individuals [Figure 4]. In this cohort, 70 families exhibited autosomal dominant inheritance of neurodegenerative diseases, as evidenced by the presence of three affected individuals in two consecutive generations. The families were later classified according to the diagnosis of the proband, with 48 families identified as having AD and 22 as FTD. These families were subsequently categorized based on the age at disease onset: ‘late onset’ was assigned to families where all affected members presented dementia at ages older than 65 years; ‘early onset’ applied to those where all affected individuals were 65 years or younger at the time of dementia onset; and ‘mixed onset’ described families that included members with both early and late-onset disease [Supplementary Table 6]. Though many of the carriers of pathogenic variants belonged to the ReDLat retrospective cohort, 14 of the recruited families were carriers of pathogenic variants, and the majority had a positive family history of neurodegeneration.

In a further analysis of families exhibiting autosomal dominant patterns, we assessed for the presence of at least one allele of *APOE* ε4. Among the families diagnosed with AD, nine werefinegative for *APOE* ε4 alleles, while 32 had had least an *APOE* ε4 carrier. We were able to determine the *APOE* ε4 allele status for more than one participant in seven families, and only four families demonstrated a co-segregation of the *APOE* ε4 allele with the illness. In contrast, when assessing the families with FTD, six werefinegative for *APOE* ε4 alleles, and in the one family where we could determine the *APOE* ε4 allele status for more than one participant, no co-segregation pattern was observed. [Figure 4]

## Discussion

This initial release of genomic data from the ReDLat cohort provides early insights into the genetic underpinnings of neurodegeneration within a Latin American population, supported by genomic analyses of established variants associated with AD and FTD. Our genetic ancestry analysis, leveraging data from the 1000 Genomes Project, revealed tricontinental admixture patterns across most regions and an East Asian component in Brazil, reflecting historical migration and admixture events. The study notably identifies a Significant prevalence of autosomal dominant inheritance patterns in AD and FTD, characterized by distinct age-of-onset categorizations, geographic distribution of genetic variants, and a stronger presence of the *APOE* ε4 allele in AD families. These patterns also include newly discovered variants in the *PSEN1* and *APP* genes for AD, which play critical roles in the disease’s pathogenesis and the recurrence of a *SOD1* variant presenting as FTD without motor symptoms, suggesting a novel disease phenotype association. Families with as-yet unidentified variation remain strong candidates for future novel gene discovery as additional family members are recruited for gene-mapping linkage studies.

Indeed, there is considerable potential for novel genetic discovery in diverse cohorts such as ReDLat, both in terms of risk for AD and related dementias (ADRD) and resilience against it, in both families and sporadic cases. Previous work in the region has unveiled more than 13 *PSEN1* pathogenic variants in Colombia, including the E280A kindred that spans more than 5000 descendants of a founder couple^11^. Leveraging larger, diverse cohorts–as well as genetic families with substantial clinical heterogeneity–represents a unique opportunity for the discovery of resilience factors for ADRD, which may serve as strong targets for disease intervention.

As previously noted by Browning et. al. (2018)^25^, historical factors like colonization, migration, and bottlenecks have Significantly shaped the genetic landscape of Latin American populations. During and after the period of colonization, many Latin Americans lived in small, often isolated villages. This created a population structure characterized by multiple mini-bottlenecks as descendants of a small number of founders in each village tended to remain in the same location for many generations. As families expanded, the specific rare alleles in each place became common, even surpassing the allelic frequencies of the same variant in the ancestral population, as is the case for the *SQSTM1* FTD risk-conferring variant Pro392Leu (rs104893941)^26–28^. This phenomenon resulted in a genetic map of the region that closely corresponds to the geographic map, where multiple individuals share the same deleterious variants and are identical by descent. The long stretches of identical haplotypes created by the bottlenecks and increased ancestral diversity within isolated populations are advantageous for researchers seeking to identify rare variants associated with diseases like AD and FTD or the interaction between genetic variation and ancestral haplotypes^29^. These factors underscore the value of family studies in Latin America, offering unique insights into genetic patterns and the potential for discovering new genetic contributions to disease^25^.

The first-wave study cohort reported here has several limitations. First, we have chosen not to conduct unbiased discovery efforts, such as genome-wide association studies and burden analyses, in this cohort due to the extensive family structure and relatively small sample size of the cross-sectional cohort collected to date. Second, despite the tangible advancement in global representation offered by this cohort, participants are still enriched for higher socioeconomic status due to the urban-centric recruitment. This drawback is being actively addressed through ongoing enrollments and community outreach efforts in more rural areas. Third, as with any clinic-based enrollment cohort, there is a possibility of ascertainment bias among recruited participants because the study recruits from clinical practices specializing in cognitive disorders, which may lead to an overrepresentation of more extreme clinical phenotypes. The findings to date represent Significant advances in understanding the etiology of Alzheimer’s and Frontotemporal dementia in this region. Continued enrollment in this project will provide additional valuable insights through future studies that map the genetic underpinnings of disease risk in large families, genetic risk burden in cases, and offer well-powered cohorts for case-control studies to identify common risk variants. Moreover, the robust family structure already observed in ReDLat provides a unique opportunity to map genetic modifiers and assess the impact of local genomic ancestry. As global population representation continues to expand, it will be critical to evaluate the generalizability of genetic risk factors for AD and FTD across diverse ancestral backgrounds, within the context of distinct social determinants of health, and accounting for modifiable risk factors that may influence disease risk and resilience across distinct cultures.

## Methods

### 1. Participant recruitment

Clinical diagnosis was determined by site investigators through consensus conferences at each site, adhering to the current diagnostic criteria for AD and FTD^30–32^. Healthy controls were recruited at the same locations meeting the following criteria: Clinical Dementia Rating (CDR)^33^ of 0, a Mini-Mental State Examination (MMSE)^34^ score greater than 25, or having been evaluated by a neuropsychologist who confirmed normal cognition in participants with few years of formal education. Family members of participants with AD or FTD, aged 18 years or older, were included if there were two or more individuals with neurodegenerative illnesses in the family, or were related to a study participant with a known dementia-associated genetic mutation and having undergone genetic counseling. All participants (diagnosed patients, healthy controls, and family members) demonstrated minimum fluency in the language of assessment (Spanish or Portuguese), had adequate vision and hearing for cognitive testing as determined by the investigator, and were required to have a study partner (informant) with at least six months of knowledge about their daily activities and cognitive/functional status. All participants (prospective and retrospective) had to be capable of providing informed consent or have a legally authorized representative.

Written informed consent was obtained from all participants or their legally authorized proxies for all evaluations and assessments conducted, following a detailed explanation of the procedures, associated risks, and potential benefits. This process adhered to the ethical guidelines of each participating country, the Code of Ethics of the World Medical Association, the Declaration of Helsinki, and the Belmont Report. Assent was also secured from participants themselves, ensuring their willingness to participate. The consent process included explicit permission to publish the findings. The study and informed consent procedures were approved by the Institutional Review Board of each participating medical institution.

Participating institutions and their respective Federalwide Assurance (FWA) numbers included the following: Argentina – INECO-Centro de Psicología Médica San Martín de Tours (FWA00028264); Brazil – Hospital das Clínicas da Faculdade de Medicina da Universidade de São Paulo (FWA00001035); Chile – Hospital Clínico Universidad de Chile (FWA00029089) and Universidad Adolfo Ibáñez (FWA00030846); Colombia – Comité de Bioética del Instituto de Investigaciones Médicas, Facultad de Medicina, Universidad de Antioquia (FWA00028864), Pontificia Universidad Javeriana - Hospital Universitario San Ignacio (FWA00001113), and Fundación Valle de Lili (FWA00029865); Mexico – Instituto Nacional de Ciencias Médicas y Nutrición Salvador Zubirán (FWA00014416); Peru – Hospital Nacional Docente Madre Niño San Bartolomé (FWA00010121); and the United States – University of California, San Francisco – Memory and Aging Center (FWA00000068).

### 2. Clinical characterization:

As part of the recruitment process, participants were interviewed about their family history of neurodegeneration. Information was collected via self-report from both patients and their study partners using the Genetic Pedigree Software-Progeny^®35^. A positive family history was defined as having at least one first- or second-degree relative with dementia or another neurodegenerative disorder. Families with three or more affected individuals in two consecutive generations were then labeled as ‘strong family aggregation’. Medical history and a full neuropsychological examination were conducted as described in Ibañez et. al.(2021)^13^ Retrospective participants were assessed based on a re-evaluation of the available clinical data for each individual. The cognitive tests were harmonized as described in Maito et.al (2023)^36^.

### 3. Genetic sequencing

Standardized phlebotomy with EDTA tubes was used for sample collection. Genomic DNA was extracted using Wizard^®^ Genomic DNA Puri cation Kit (Promega), QIAamp^®^ DNA Mini Kit (Qiagen), or similar salting-out methods. Samples were shipped quarterly from the various participating sites in Latin America to the United States. HudsonAlpha Institute for Biotechnology (Alabama, U.S.A) performed Single Nucleotide Polymorphism (SNP) Arrays, whole exome sequencing (WES), and/or whole genome sequencing (WGS) of the samples. Additional whole genome sequencing was performed at Psomagen, Inc. (Maryland, U.S.A)

Variants were genotyped using the NeuroBooster array from Illumina, designed to capture variants relevant to neurological conditions^37^. Quality control (QC) of the SNP Array data was conducted using Genotools v1 default settings^38^. Prior to imputation, the QC’ed output files from Genotools were processed with the *no_qc_imputation_prep.sh* script. This script ran the datasets through the Wrayner script to compare them against all TOPMed freeze 8 variants. Excluded variants were then ipped to rescue additional variants, after which the dataset was processed again through the Wrayner script to compare against PASS TOPMed freeze 8 variants. Data was subsequently imputed using the TOPMed Imputation Panel and Server v1.3.3, following a previously developed pipeline for multi ancestral sample sets as described in Vitale et. al. (2024)^38^.

For WES, DNA was processed using Integrated DNA Technologies xGen Exome Hyb Panel v2, and sequenced on the NovaSeq 6000 platform using paired-end 100-base pair reads to a target depth of 100X.

WGS was performed at two institutions; Samples at Psomagen were prepared using the TruSeq DNA PCR-Free library prep method to avoid PCR amplification bias. Samples at HudsonAlpha (64 of the pass-QC genomes) underwent a custom PCR-free preparation involving Covaris shearing (fragmenting the DNA), end repair (preparing the DNA fragments for ligation), and adapter ligation, all without PCR amplification. All libraries from both sites were then normalized using KAPA qPCR and sequenced on the Illumina NovaSeq 6000 platform to a target depth of 30X. The sequencing was paired-end with a read length of 150 bp (Illumina 150bpPE).

### 4. Genetic data processing

The raw sequence data (fastq files) were aligned to the hg38 reference genome using the Sentieon v202112.05 implementation of the BWA MEM algorithm at HudsonAlpha. Sentieon v202112.05 utilities were used to sort the reads, mark duplicate sequences, and recalibrate the base quality scores. Variant calling, which identifies differences between the sample DNA and the reference genome, was performed using GATK4 tools implemented by Sentieon v202112.05. This step was conducted across all samples in a batch for exomes and one for genomes to maintain consistency. Finally, variant quality score recalibration (VQSR) was applied to filter out false positive variant calls, ensuring high-quality data. This comprehensive approach achieved an average recall rate of 99.22% when compared to the Genome in a Bottle high confidence truth sets, indicating a high level of accuracy in detecting genetic variants.

Variant Call Format (VCF) les were filtered according to established criteria to ensure high-quality data. For whole genome and exome sequences variants with genotype quality greater than 20 and read depth scores above 10 were retained. The filtered VCF was then annotated with gene names, variant types, and amino acid changes for all exonic variants using GRCh38.99 with SnpEff, dbSNP release 156, CADD 1.6, TOPMed Bravo Freeze 8 allele frequencies, and ClinVar through BCFtools and Annovar^39–43^. Variants with genotyping rates below 95% by individual and 95% by variant were removed. Chromosomal sex was further validated via genetic data by splitting the pseudoautosomal regions of the X chromosome and analyzing the heterozygosity of X-chromosome, as well as the count of variants present on the Y chromosome. A detailed pipeline and scripts are available at https://github.com/TauConsortium/redlat-genetics

To combine the arrays and WGS data, all variant IDs in both datasets were first annotated with “chrom:pos:ref:alt”. The VCFs were then intersected using BCFtools v1.9 isec, producing a list of intersecting variants between the two datasets. Both VCFs were filtered to include only these intersecting variants and then merged using BCFtools v1.9 merge^45^ After merging, the VCF was annotated with dbSNP 156^46^.

To ensure concordance between the imputed arrays and WGS, concordance was checked for individuals with data from both methods. The imputed array data was filtered for varying allele frequencies (AF) using BCFtools v1.9^45^. Concordance was assessed for each AF-filtered VCF using SnpSift concordance. The final concordance for each individual was determined by dividing the number of correct variant calls by the total number of intersecting variant calls. The complete script for the concordance check is available at the project’s GitHub repository [see “Code Availability”]. Samples with concordance below 0.95 at sites with AF less than 0.0001 were excluded.

### 5. Genetic Relatedness

Family history was documented through elaboration of detailed pedigrees for each recruited participant. Disclosed relatedness was compared to expected genetic relatedness using KING^44^. Individuals with cryptic relatedness (kinship coefficient <0.125 without a familial relationship documented on the pedigrees) or discrepancies between disclosed and genetic relatedness were removed.

### 6. Population stratification

To capture the ancestral diversity of the cohort and represent all participating countries, we merged the WGS data with imputed SNP array data for individuals lacking WGS data. The ReDLat dataset was then combined with high-depth WGS data from the 1000 Genomes Project (1000GP)^47^ and filtered to include only biallelic variants with a genotyping rate of >95%. The resulting ReDLat-1000GP dataset was used for the following analyses:

Principal component analysis (PCA) was conducted using the smartpca package from EIGENSOFT (version 8.0.0)^48^ PCA was performed on samples from 1000GP, and the ReDLat samples were subsequently projected onto the principal components.

Global ancestry was estimated using ADMIXTURE (version 1.3)^49^. We performed an unsupervised ancestry analysis on the 1000GP data, modeling ancestry from two to eight populations (K). The ancestry fractions for the ReDLat samples were calculated using the allelic ancestry proportions derived from the 1000GP analysis.

### 7. Variant pathogenicity analysis

Data from WGS and WES was merged for a joint analysis for pathogenic variation. We used the Online Mendelian In Men (OMIM) database to search for genes associated with autosomal dominant, autosomal recessive, or X-linked forms of adult-onset dementia^50^.

We manually curated protein-altering variants in the ten genes most commonly associated with adult-onset neurodegeneration: *APP*, *CHMP2B*, *FUS*, *GRN*, *MAPT*, *PSEN1*, *PSEN2*, *TARDBP*, *TBK1*, and *VCP*, as well as expansions in *C9orf72* (identified using ExpansionHunter v5.0.0)^51,52^. Variants located in introns, the 3′ untranslated region (3′ UTR), the 5′ untranslated region (5′ UTR), and synonymous variants within exons were included if their in silico splice-predicting scores (dbscSNV_RF_SCORE and dbscSNV_ADA_SCORE) were both greater than 0.6, since these variants were considered likely to have an impact on splicing, making them relevant for further study^53^. Exonic non-synonymous variants (missense, nonsense, and frameshift) were analyzed following guidelines from the American College of Medical Genetics and Genomics (ACMG)^52^ and the Guerreiro algorithm for *PSEN1* and *PSEN2* genes^5^. The variants identified in the remaining genes listed in Supplementary Table 3 were queried in ClinVar^54^ databases and reported if previously classified as pathogenic or likely pathogenic. Variants identified through this process were then tested by hand for familial segregation to con rm their association with the disease within families.

### 8. Local ancestry of pathogenic variants

To determine the haplotypic origin of pathogenic variants, we first phased the ReDLat WGS using SHAPEIT5 (v5.1.1)^55^. Pedigree information and 1000GP phased genomes were included as family and population references to improve phase accuracy. Since heterozygous variants in a single individual cannot be reliably assigned to a specific haplotype, we restricted our assessment of local ancestry to pathogenic variants present in two or more individuals.

After phasing, we estimated the local ancestry of pathogenic variants using RFMix (v2.03-r0)^56^. We constructed the reference population panel by merging high-coverage sequence data from the Human Genome Diversity Project (HGDP)^57^ and 1000GP. Due to the absence of Amerindian individuals in the 1000GP dataset, we used admixed American samples from 1000GP that were over 99.9% Amerindian in the ADMIXTURE analysis at K=3 as Amerindian reference samples. We then extracted representative individuals of African, Amerindian, European, and East Asian ancestry to build reference cohorts of similar size (100–150 individuals). Finally, we divided the ReDLat WGS into subgroups of individuals with similar global ancestry based on their ADMIXTURE results and ran RFMix with the following settings: terminal node size of five, five expectation-maximization iterations, and both with and without the --reanalyze-reference option.

## Figures and Tables

**Table 1.** Clinical characteristics of included ReDLat participants

		AD n=999	FTD n=381	MCI n=8	OTHER n=19	HC n=755
**Age<65years, n (%)**		139 (13.9)	85 (22.3)	2 (25.0)	2 (10.5)	277 (36.7)
**Female, n (%)**		665 (66.6)	201 (52.8)	6 (75.0)	8 (42.1)	516 (68.3)
**AD Phenotype, n (%)**	Amnestic	521 (52.2)	-	-	-	-
	lvPPA	10 (1.0)	-	-	-	-
	PCA	6 (0.6)	-	-	-	-
	fvAD	1 (0.1)	-	-	-	-
	usAD	461 (46.1)	-	-	-	-
**FTD Phenotype, n (%)**	bvFTD	-	196 (51.4)	-	-	-
	nfPPA	-	25 (6.6)	-	-	-
	svPPA	-	37 (9.7)	-	-	-
	FTD-CBS	-	26 (6.8)	-	-	-
	FTD-MND	-	9 (2.4)	-	-	-
	FTD-PSP	-	9 (2.4)	-	-	-
	usFTD	-	72 (18.9)	-	-	-
	usPPA	-	7 (1.8)	-	-	-
***APOE*ε4 carriers, n (%)**	Heterozygous	408 (41.5)	96 (25.9)	1 (14.3)	2 (10.5)	153 (20.7)
	Homozygous	85 (8.6)	15 (4.0)	-	1 (5.3)	13 (1.8)
**Country of Origin**	Argentina	191 (19.1)	2 (0.5)	-	1 (5.3)	74 (9.8)
	Brazil	42 (4.2)	99 (26.0)	-	-	88 (11.7)
	Chile	115 (11.5)	45 (11.8)	6 (75.0)	11 (57.9)	115 (15.2)
	Colombia	536 (53.7)	193 (50.7)	1 (12.5)	6(31.6)	289 (38.3)
	Mexico	59 (5.9)	17 (4.5)	-	-	133 (17.6)
	Peru	56 (5.6)	25 (6.6)	1 (12.5)	1 (5.3)	56 (7.4)
**Genomic Data, n (%)**	SNP Array	282 (28.2)	126 (33.1)	6 (75.0)	10 (52.6)	234 (31.0)
	WES	6 (0.6)	-	-	-	3 (0.4)
	WGS	711 (71.2)	255 (66.9)	2 (25.0)	9 (47.4)	518 (68.6)

AD: Alzheimer’s disease, FTD: frontotemporal dementia, MCI: mild cognitive impairment, HC: healthy controls, n: number, lvPPA: logopenic variant primary progressive aphasia, PCA: posterior cortical atrophy, fvAD: frontal variant AD, usAD: unspecified Alzheimer’s disease, bvFTD: behavioral variant frontotemporal dementia, nfPPA: nonfluent variant primary progressive aphasia, svPPA: semantic variant primary progressive aphasia, FTD-CBS: frontotemporal dementia-corticobasal syndrome, FTD-MND: frontotemporal dementia-motor neuron disease, FTD-PSP: frontotemporal dementia-progressive supranuclear paralysis, usFTD: unspecified frontotemporal dementia, usPPA: unspecified primary progressive aphasia. SNP: single nucleotide polymorphism, WES: whole exome sequencing, WGS: whole genome sequencing

**Table 2.** Pathogenic variants found in primary AD/FTD genes.

Gene	Coding Change	AA Change	ExAC	CADD	REVEL	Family history	ACMG Classification	Country of origin	Proband's Phenotype(s)
***APP***	c.2020G>C	Glu674Gln	< 0.00001	25.2	0.635	A-Dom	Likely pathogenic	Mexico	Amnestic AD
***C9orf72***	C.-45+163 GGGGCC [>24]		.	.	.	A-Dom	Pathogenic	Chile	bvFTD
								Brazil	
***GRN***	c.21G>A	Trp7Ter	.	36	.	Sporadic	Pathogenic	Colombia	usAD
	c.58dupT	Cys20Leufs*45	.	.	.	A-Dom	Pathogenic		bvFTD
								Chile	
	c.328 C>T	Arg110Ter	< 0.00001	29.4	.	Positive	Pathogenic	Colombia	FTD-PSP
	c.462+1 G>A	.	.	26.3	.	Positive	Pathogenic	Peru	bvFTD
	c.767_768insCC	Gln257Profs*27	.	.	.	Unknown	Pathogenic	Brazil	bvFTD
	c.1098 T>A	Cys366Ter	.	36	.	Positive	Pathogenic	Colombia	svPPA
***MAPT***	c.796 C>G	Leu266Val	.	25.8	0.662	A-Dom	Pathogenic	Brazil	bvFTD
	c.915 T>C	Ser305Ser	.	.	.	A-Dom	Pathogenic	Colombia	bvFTD
	c.1280 C>T	Thr427Met	0.00001	33	0.508	Unknown	Pathogenic	Argentina	Amnestic AD
***PSEN1***	c.250 A>G	Met84Val	.	23.9	0.923	Unknown	VUS	Argentina	Amnestic AD
	c.280 G>A	Val94Met	< 0.00001	25.1	0.847	A-Dom	Pathogenic	Colombia	usAD
	c.356 C>T	Thr119Ile	.	24.4	0.795	A-Dom	Pathogenic	Colombia	bvFTD
								Argentina	
	c.415 A>G	Met139Val	.	23.4	0.879	A-Dom	Pathogenic	Argentina	Amnestic AD
	c.428 T>C	Ile143Thr	.	26.8	0.985	A-Dom	Pathogenic	Colombia	Amnestic AD
	c.519 G>T	Leu173Phe	.	25.8	0.923	A-Dom	Pathogenic	Colombia	bvFTD
	c.1223 T>C	Ile408Thr	.	27.4	0.877	Unknown	VUS	Peru	Amnestic AD
***VCP***	c.283 C>T	Arg50Cys	.	27.1	0.606	A-Dom	Pathogenic for essential tremor	Colombia	Amnestic AD
***TARDBP***	c.1147 A>G	Ile383Val	< 0.00001	0.308	0.377	A-Dom	Pathogenic	Colombia	usFTD

AA: Amino acid. A-Dom: Autosomal dominant; three affected individuals in two generations. Positive family history; At least one first or second degree relative with neurodegeneration. usAD: Unspecified Alzheimer's disease, bvFTD: behavioral variant frontotemporal dementia, FTD-PSP: frontotemporal dementia-progressive supranuclear paralysis, svPPA: semantic variant primary progressive aphasia, AD: Alzheimer's disease, VUS: Variant of unknown significance. PVS1: The PVS1 rule is related to null variants which usually result with a loss of function effect. These variants include stop gain, frameshift insertions or deletions, splice donor or acceptor, a start loss

**Table 3.** Ancestral origin of pathogenic variants found in more than one carrier

Gene	Coding Change	AA Change	Number of independent families	Number of Carriers with WGS/WES	Ancestral Haplotype
***GRN***	c.767_768insCC	Gln257Profs*27	2	2	European
	c.1098 T>A	Cys366Ter	1	3	European
***MAPT***	c.915 T>C	Ser305Ser	1	3	European
***PSEN1***	c.356 C>T	Thr119Ile	4	16	European
	c.415 A>G	Met139Val	1	8	Amerindian
***TARDBP***	c.1147 A>G	Ile383Val	1	2	European

AA: Amino acid. WGS: whole genome sequencing, WES: whole exome sequencing. The 'number of independent families' clusters individuals into 'families' based on pedigree reconstruction from the participant interview.

## Data Availability

Data used in this publication has been uploaded to the FAIR Data Portal within the AD Workbench, which is supported by the Alzheimer’s Disease Data Initiative, and can be accessed at https://www.alzheimersdata.org/ad-workbench.
